# Rice Bran Extraction and Stabilization Methods for Nutrient and Phytochemical Biofortification, Nutraceutical Development, and Dietary Supplementation

**DOI:** 10.1093/nutrit/nuae174

**Published:** 2024-12-06

**Authors:** Prajna Priyadarshini Das, Mir Zahoor Gul, Annika M Weber, Rakesh K Srivastava, Balram Marathi, Elizabeth P Ryan, Irfan A Ghazi

**Affiliations:** Department of Plant Sciences, School of Life Sciences, University of Hyderabad, Hyderabad, Telangana 500046, India; Department of Plant Sciences, School of Life Sciences, University of Hyderabad, Hyderabad, Telangana 500046, India; Department of Food Science and Human Nutrition, Colorado State University, Fort Collins, CO 80523, United States; Genomics, Pre-breeding, and Bioinformatics (GPB), Accelerated Crop Improvement (ACI), International Crops Research Institute for the Semi-Arid Tropics (ICRISAT), Patancheru, Telangana 502324, India; Department of Genetics and Plant Breeding, Agricultural College, Warangal, Telangana 506007, India; Department of Environmental and Radiological Health Sciences, Colorado State University and Colorado School of Public Health, Fort Collins, CO 80523, United States; Department of Plant Sciences, School of Life Sciences, University of Hyderabad, Hyderabad, Telangana 500046, India

**Keywords:** rice bran, stabilization, extraction, functional foods, biofortification, nutraceutical development, dietary supplementation

## Abstract

Rice is a global staple food crop for nearly half of the world's population. Rice bran along with the germ are essential components of whole-grain rice and have immense potential for enhancing human nutrition. Rice bran has a unique composition and distinct requirements for processing before it can be consumed by humans when compared with other cereal brans. The comprehensive overview and synthesis of rice bran processing include extending the shelf life for functional food product development and extraction of bioactive components. This narrative review highlights established and innovative stabilization approaches, including solvent extraction and enzymatic treatments, which are critical methods and technologies for wider rice bran availability. The nutrient and phytochemical profiles of rice bran may improve with new cultivar development and food-fortification strategies. The postharvest agricultural practices and processing techniques can reduce food waste while also supporting growers to produce novel pigmented cultivars that can enhance nutritional value for human health.

## INTRODUCTION

Rice, which originates from the seeds of the monocotyledonous plant *Oryza sativa* L. (Asian rice) and *Oryza glaberrima* (African rice), is a significant cereal crop for the world as a staple food for more than half of the global population. Rice has long been regarded as a vital food dating back to before written history, and whole-grain rice is gaining attention for its functional human health properties. Rice is cultivated in over 114 nations across the globe and is forecast to grow by 1.4%, leading to an increase in food-consumption trends for cereals. Higher rice inventories are also expected to contribute to a record 897 million tonnes in world cereal stocks. Contrary to trends in maize and wheat, the international rice trade is forecast for robust growth.^1^

During harvest, the rice kernel is completely encased by the rice hull. A white, carbohydrate-rich endosperm kernel is encircled by an adherent bran cover that is wrapped in an outer casing referred to as a hull or husk. Rice grains pass through many processing stages postharvest and before it is utilized as food. The steps include cleaning, hulling, and post-hulling processing (polishing, whitening, and grading). The rice milling procedure removes the husk and bran coverings (layers) from paddy rice, resulting in an edible white, carbohydrate-rich endosperm kernel. After the husk is removed, the bran layer is typically milled off, leaving white rice. Notably, the rice germ is contained in the rice bran after polishing. Bran accounts for approximately 7%–10% of the total weight of whole-rice grain.^2^ Rice bran usage is limited and is not generally consumed by humans since it is not collected with safety precautions and may include broken grains or hull contaminants from milling.^3^ Further, the enzyme lipase will degrade the rice bran oils (RBOs) and render the bran rancid and inedible with an unfavorable aroma after polishing. Due to rice bran's high-fat content, rice bran has a relatively short shelf life in the absence of heat stabilization. Because of this, 90% of the rice bran harvested annually is used to make feed for poultry and livestock, and the remaining 10% is utilized for the production of RBO.^4–6^ Rice bran remains available at a low cost. It has high value due to its antioxidant potential and promising effects against several metabolic ailments.^7^^,^^8^ For example, rice bran has been effectively incorporated into a variety of food items, such as oil, bread, pastries, pasta, noodles, beverages, and sweet frozen foods, without adversely impacting the textural and functional properties.^5^^,^^8^^,^^9^ This review article details the various approaches for processing rice bran and its bioactive food components for optimal utility, including by solvent extraction, enzymatic treatments, and innovative stabilization methods before inclusion in food and related supplement products.

## RICE BRAN AND PIGMENTED RICE

**Figure 1.**
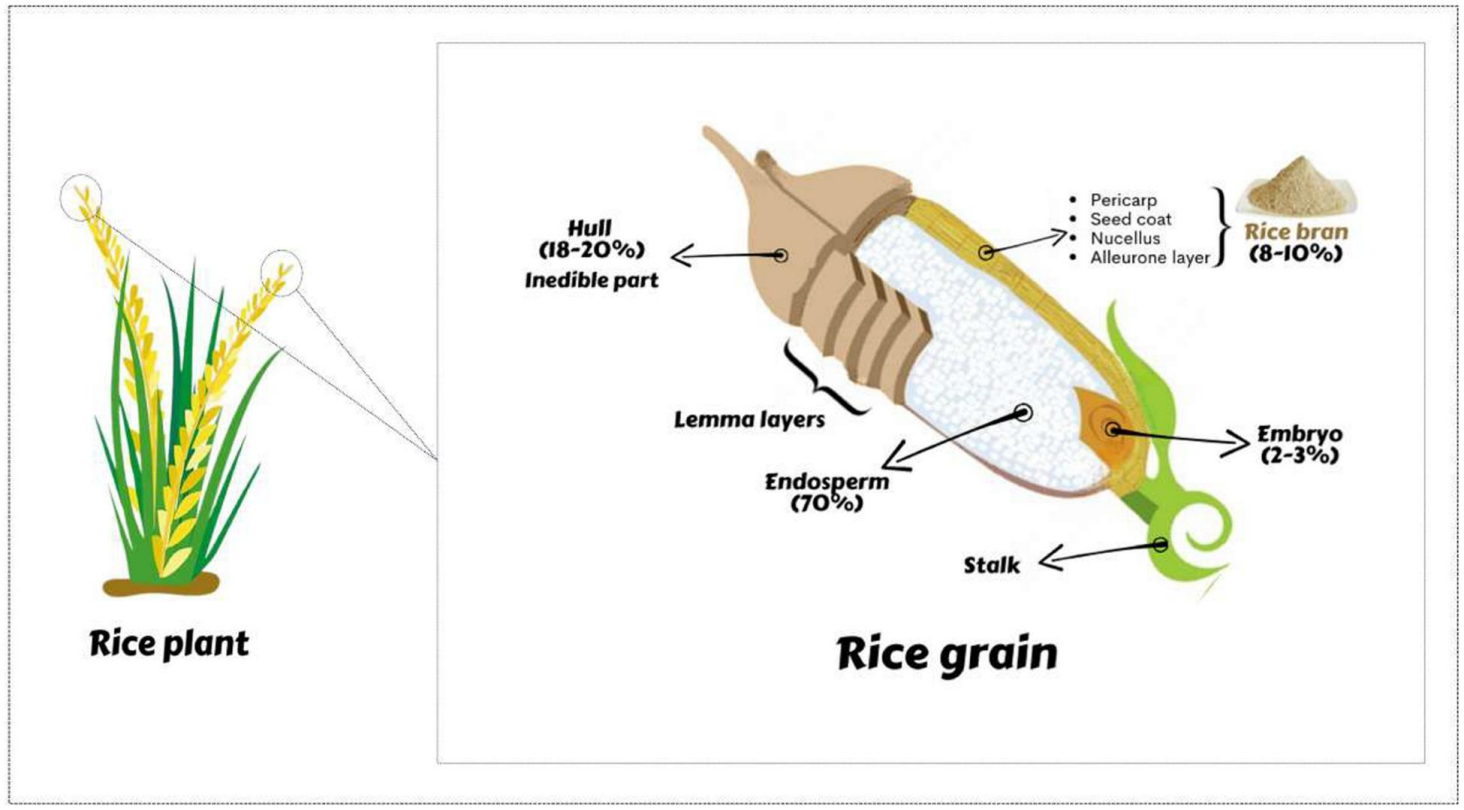
Visualization of the Structural Organization of the Rice Grain. The figure illustrates the rice grain's structural organization, highlighting its key components and their respective layers

Rice bran is the brown outer layer of the rice kernel, mainly composed of the pericarp, aleuron, seed coat, and germ, and it is separated during the milling process. **Figure 1** illustrates the intricate structural organization of a rice kernel, detailing its various components and layers including bran. Rice bran, which makes up 8%–10% of the total grain, is a rich source of vital nutrients and bioactive constituents, such as 11%–17% of protein, 12%–22% of fat, 10%–15% of moisture, 6%–14% of fiber, and 8%–17% of ash.^10^^,^^11^ The endosperm mainly contains carbohydrates, whereas the proteins are present in the rice bran and germ layers along with minerals, vitamins, and dietary fiber; and vitamins, minerals, amino acids, protein, and lipid bodies are rich in the aleurone layer. Many cultivars of rice contain colored pigments, and these pigments are generally localized in the pericarp (aleurone layer) or the bran of the rice kernel, which results in different colors of rice.^12^^,^^13^ The pigmented rice mainly comprises various mixtures of colors, such as black, dark-purple, brown, dark brown, and red, and red-grain rice,^14–19^ which completely depends upon the kinds of pigment colors. Certain traditional landraces accumulate higher phytochemicals in rice grain than rice grain produced by commercial varieties.^20^ Pigmented rice has drawn great attention in the past few years due to its nutritional value, which is largely conferred by its abundant content of diverse bioactive compounds like anthocyanins, flavones, tannins, phenolics, tocols, sterols, γ-oryzanols, amino acids, and essential oils, etc, and exhibits many bioactivities including antioxidant and free radical scavenging, antitumor, anti-atherosclerosis, anti-hypoglycemic, and anti-allergic activities that promote health benefits.^8^^,^^9^^,^^21^ Rice bran was found to contain ∼450 known metabolites.^22^ However, many factors influence the composition of rice bran, such as rice varieties, geographical region of production, agricultural practices, and postharvest milling process. Overall, these compounds make rice bran a valuable addition to the diet, offering a significant source of bioactive compounds that promote overall well-being and disease prevention when incorporated into meals.

Understanding the stability of these pigments during processing is crucial for maintaining their efficacy and visual appeal in end products. Furthermore, the diverse range of pigmented rice varieties, each with its unique color profile, presents promising opportunities for culinary and industrial applications. Additionally, traditional landraces, which accumulate higher phytochemical content compared with commercial varieties, show the potential for enhanced nutritional value and health benefits.^23^ The compounds present i+n pigmented rice—notably, anthocyanins, flavones, flavonols, and phenolic components—play a pivotal role in enhancing its color stability, thereby expanding its applications in various food products. Anthocyanins, responsible for the red, purple, and blue hues in rice bran, act as natural pigments that resist fading or degradation during processing and storage. Similarly, flavones, flavonols, and phenolic components, such as ferulic and *p*-coumaric acid, contribute to the color stability of rice bran by protecting against oxidation and enzymatic browning, preserving the vibrant colors of rice bran–based foods and extending their shelf life. These compounds not only enhance the visual appeal of rice bran–based products but also broaden their potential applications in cereals, baked goods, snacks, and beverages, where color stability is essential for consumer satisfaction and product quality.^21^^,^^24^^,^^25^

## RICE BRAN STABILIZATION: RATIONALE AND IMPORTANCE

Rice bran is an emerging novel food ingredient that provides numerous beneficial effects to the global population. With the ever-increasing demand for nutritious and sustainable food options, rice bran has gained popularity in the food industry. Rice bran is rich in dietary fibers, vitamins, minerals, and bioactive compounds. It provides a wide range of health-promoting properties that include the ability to enhance metabolism, improve immune function, prevent cancer, protect cardiovascular disease, and reduce the risk of chronic diseases.^8^ As the world recognizes the importance of balanced diets, it is crucial to highlight the benefits of rice bran, an underutilized food waste, especially to meet nutritional requirements for the ever-increasing global population.

Although rice bran contains a rich variety of nutrients, a major drawback is the short shelf life due to its high susceptibility to rancidity. Rice bran contains lipids (15%–20% oil), and high activities of lipases and lipoxygenases result in the lipid becoming easily rancid, leading to a decline in nutritional value.^26^ This happens when the bond between fatty acid and glycerol ester is broken by lipases, and free fatty acid (FFA) production increases within hours of milling. Therefore, rice bran is mainly used as livestock feed or boiler fuel, and only a small amount is applied to extraction and preparation of RBO.^27^ Stabilization techniques are crucial to slow down lipase activity and preserve the valuable nutritional resource for consumption, allowing rice bran to be stored for approximately 6 months. As the world recognizes the importance of balanced diets, the demand for rice bran as an emerging ingredient in the food industry is increasing, highlighting its potential for meeting nutritional requirements and promoting sustainable food options.^8^^,^^28^^,^^29^ To improve the utilization of rice bran, the development of feasible stabilization methods and the value-added processes of the nutrients and active compositions are necessary. The ideal stabilization process aims to maintain the continuity of rice bran's nutritional properties over time by reducing lipase activity.^30^^,^^31^

Bran stabilization refers to the treatment process to inhibit enzymatic activity that leads to rancidity. The stabilization techniques help slow down the lipase activity within the bran and preserve the valuable nutritional resource for consumption. Stabilized rice bran can typically be stored for approximately 6 months, particularly in the winter season. Therefore, stabilization procedures are extremely necessary for human consumption of rice bran.^30^ The ideal stabilization process will maintain the continuity of the rice bran's nutritional properties over time by reducing the lipase activity to the lowest possible level.^31^ In most cases, the stabilization process helps in increasing nutritional value, which is beneficial for further food processing.^28^ Hence, stabilization is indeed necessary to prevent rancidity, thereby extending the shelf life of bran and maintaining its nutritional quality.^32^

### Rice Bran Stabilization Techniques

**Figure 2.**
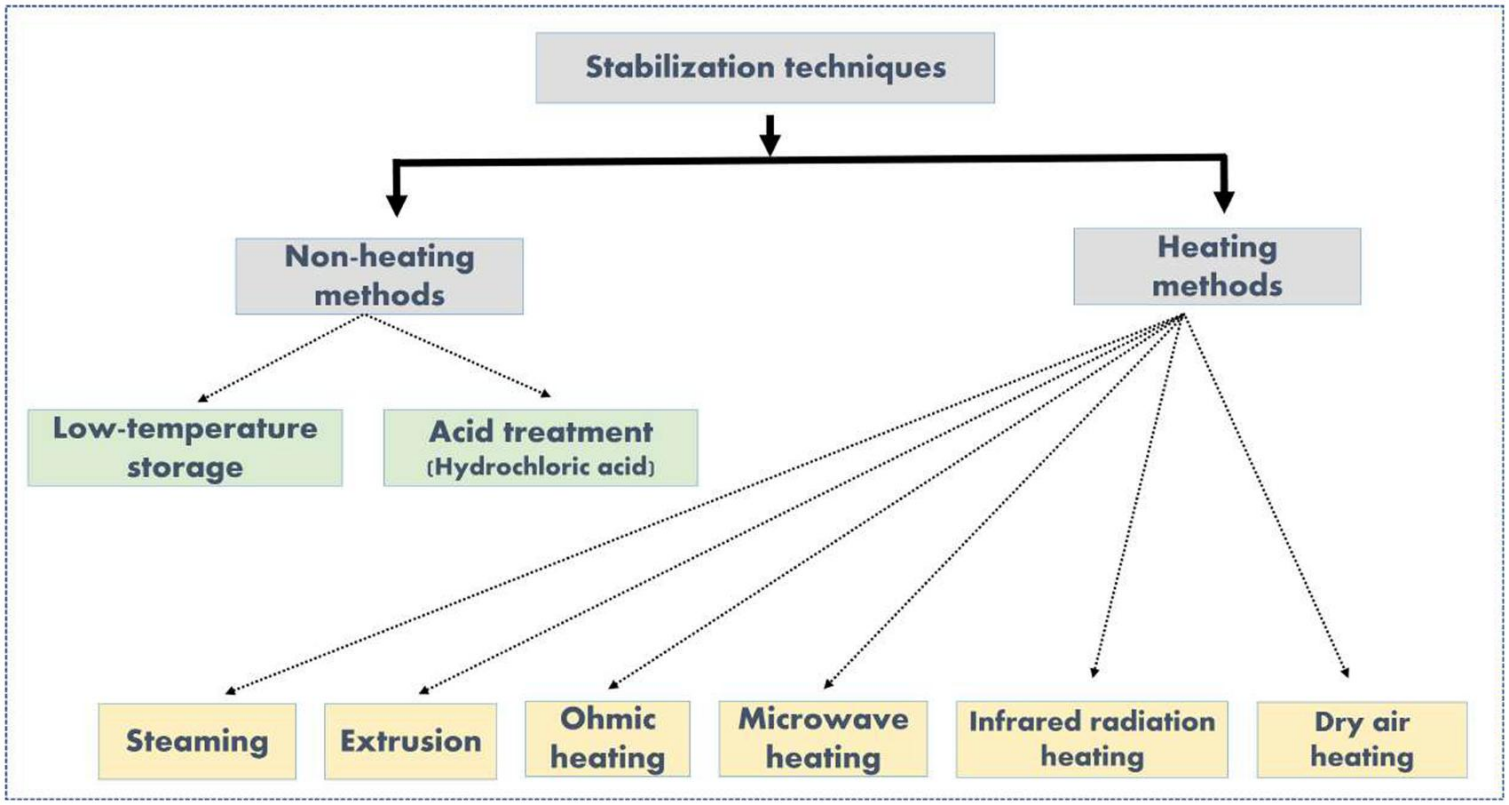
Rice Bran Stabilization Techniques. The figure illustrates the various methods used for the stabilization of rice bran, organized into non-heating and heating approaches. These methods are essential for extending the shelf life of rice bran and ensuring its effective utilization in food products

Less than 5% of FFA content is suitable for the human diet, but above this value the product is unfit for human consumption.^33^ To store the rice bran for an extended period, rancidity must be controlled. Therefore, rice bran must undergo stabilization to reduce the lipase enzymatic activity without altering its nutritional resources. There are several rice bran stabilization processes, which include ohmic heating, acid treatment, dry heating, microwave heating, infrared heating, etc. **Table 1**^28^^,^^31^^,^^34–53^ presents various stabilization techniques used for rice bran, outlining their specific conditions, underlying mechanisms, and resulting nutritional outcomes. Furthermore, **Figure 2** presents a comparative visual representation of various stabilization techniques used in preserving rice bran, categorized into non-heating and heating approaches. The figure aims to provide a comprehensive overview and comparison of non-heating and heating stabilization techniques for rice bran, emphasizing their respective methodologies.

**Table 1.** Stabilization Techniques With Distinct Conditions, Mechanisms, and Nutritional Outcomes

Reference	Stabilization techniques		**Standardized** **conditions of stabilization**	Mechanisms of lipase inactivity	Inference or finding	Pros	Cons
Amarasinghe et al (2009)^34^	Non-heating process	Low-temperature storage	0°C	Lipase activity reduced at 0°C	FFA content was reduced during the storage period. FFA increased in room temperature due to enzymatic activation. Requires high energy consumption for maintaining low-temperature conditions for storage.	Cold storage protects the nutritional value. Preserves oils, vitamins, and antioxidants. Ideal for food and animal feed industries.	Time-consuming method. Potentially increases operational costs. Increases storage needs. Challenge for industries with tight production schedules.
Akhter (2015)^35^		Acid treatment (HCl)	Acid (HCl) treatment (30 mL/kg) at room temperature	Treatment with acid helped in lipase inactivation	% of FFAs is reduced	Improves nutrient availability. Enhances nutritional value.	Environmental and safety concerns. Increased operational costs.
Lv et al (2017)^36^	Heating process	Dry air heating	120°C for 10–60 min	Inactivation of lipase activity due to controlled temperature and moisture	Increase in FFA levels in RBO is minimal or slow over 3 mo of storage. Protein and oil slightly increased. Increase in oil and water holding capacity; protein solubility destroyed.	Efficient moisture removal. Extends shelf life. Preserves nutritional quality. Gently preserves its nutritional value. Suitable for diverse applications in food and feed industries.	Can cause nutrient loss. Requires careful controlled process. Energy-intensive. Relies on electricity, natural gas, or other fuels. Higher production costs and environmental impact. Requires optimization of energy usage.
Thanonkaew et al (2012)^37^		Hot air heating	150°C for 10 min, roasting at 150°C for 2 min, and steaming at 130°C for 2 min	High temperature inhibited the lipase activity	FFAs, AV, and PV were lower than in raw rice bran		
Brunschwiler et al (2013)^38^		Dry Heat	20% moisture at 110°C for 5 min	Inactivation of lipase/esterase activity due to controlled temperature and moisture			
Gul et al (2015)^39^		Steaming	93°–104°C for 5–10 min	Denaturation of lipase activity due to steaming at set temperature	No destruction of nutritional value. Oil content decreased during the storage period.		
Randall et al (2006)^40^		Extrusion	130°C and holding for 3 min at 97°C before cooling	High temperature, pressure, and shearing force produced to inactivated enzymes (ie, lipase and peroxidase)	FFA decreased after the extrusion process. Retained nutritional value. Less time-consuming, unique and simple operating method for manufacturing products.	Enhances nutritional availability. Favorable for rice bran preservation. Improves digestibility and nutrient absorption. Valuable for animal feed and food applications.	High energy and equipment costs.
Rashid et al (2023)^41^		Extrusion	Varying temperatures (15°, 25°, and 40°C) over 60 days	Altered lipase and other enzymatic activities after stabilization	Significant changes in RB storage stability, lipid oxidation, enzymatic activity, fatty acid composition, and protein characteristics		
Rafe and Sadeghian (2017)^42^		Heat and centrifuge	123°C and centrifugation speed of 15 g	Optimized temperature and centrifugation speed improved inhibition of lipase activity	Enhanced color, boosts dietary fiber, while preserving key vitamins. Phytic acid and vitamin E levels are reduced.		
Lakkakula et al (2004)^28^		Ohmic heating	20 A voltage for 3 min	Heat energy generated inactivated the lipase enzyme	Accumulation of FFAs is very slow. Successfully developed and used in industry.	Efficient. Prevents nutrient loss and maintains nutritional value. Operates without heat transfer medium. Utilizes applied electricity. Demonstrates rapid and uniform energy conversion.	High initial costs.
Moreno et al (2021)^43^		Heat	100°C for 15 min	Heating inhibited the lipase activity and reduced FFA formation	Suitable for industrial purposes (FFA <5%) as edible oil extraction. Lipase activity was reduced (< 0.1 IU per gram of RB)		
Irakli et al (2018)^31^		Infrared (IR) heating	140°C for 15 min	Radiation energy helped in lipase inactivation by converting it into heat energy	Nutrient source retained without loss of γ-oryzanol, fatty acid composition. Vitamin E content is decreased.	Preserves nutritional content. Minimizes high-temperature exposure. Energy-efficient.	High initial equipment costs.
Wang et al (2017)^44^		Infrared (IR) heating	60°C for 4–5 h	Inactivation of the lipase activity due to long hours of temperature treatment	Reduced FFA content (<10%) for 7 days without altering the RBO quality		
Yılmaz et al (2014)^45^		Infrared (IR) heating	600 W IR power for 5 min	Reduced the activation of lipase due to high-IR power	Maintained FFA content below 5% for 165 days in the stabilized rice bran		
Kreungngern et al (2021)^46^		Infrared (IR) heating	IR power at 999 W for 562 s, and vacuum pressure with 650 mmHg	Optimized technique of stabilization under specific conditions reduced lipase activity	IR-vacuum technique showed optimal stabilization and reduced the FFA content to below 5% and maintained stability		
He et al (2020)^47^		Infrared (IR) heating	300°C for 210 s	High temperature for specific time reduced lipase and peroxidase activity	IR heating reduced lipase activity by 73.05% and peroxidase activity by 81.50%		
Lavanya et al (2019)^48^		Microwave heating	925 W for 3 min	Lipase, peroxidase, and other antioxidant enzyme inactivation due to higher microwave power for a longer exposure	FFA as well as acid and peroxide values were found to be low during the storage period because of the high power of heating	Offers quick, uniform heating. Preserves nutritional value. Minimizes exposure to high temperatures. Energy-efficient. Enhances rice bran's nutritional value.	High initial equipment costs. Potential product variations.
Pokkanta et al (2019)^49^		Microwave heating	130–880 W for 0.5–5.0 min and 440 W for 2.5 min	Optimized parameters inhibited the lipase activity and increased the nutritional value	Significantly increased the content of bioactive constituents such as total phenolics, total flavonoids, total tocols, γ-oryzanols, phytosterols, trans-p-coumaric acid, and kaempferol		
Pongrat and Songsermpong (2019)^50^		Microwave heating	6400 W, 2450 MHz for 15.75 min	High power reduced the lipase and peroxidase activity	Continuous microwave heating effectively stabilizes rice bran by reducing FFA, PV, and TBA values when stored at 4°C and 25°C for 16 wk		
Chen et al (2029)^51^ and Seyoum et al (2022)^52^	Fermentation process	Fungi, yeast, and bacteria fermentation	37°C to 43°C for 12 h to 24 h	Fungi, yeast, and bacteria produce enzymes such as amylase, β-glucosidase, and protease that helped to change the rice bran constituents	RB constituents’ changes to other metabolites, such as sugar, alcohols, organic acid, amino acids, antioxidant and vitamin compounds, improve the nutrient bioavailability and food quality for health.	Enhances the nutritional profile. Breaks down anti-nutritional factors. Improves digestibility. Produces beneficial compounds.	Time-consuming, Challenges in controlling fermentation conditions Potential product variability. Need for specific microbial strains. Challenges for industrial implementation.

Adapted and modified from Gul et al (2015)^39^ and Liu et al (2019).^53^

Abbreviations: AV, acid value; FFA, free fatty acid; HCl, hydrochloric acid; IR, infrared; PV, peroxide value; RB, rice bran, RBO, rice bran oil; TBA, thiobarbituric acid.

#### Non-Heating Process

##### Cold temperature treatment

Cold temperature treatment involves storing rice bran at temperatures below 3°C. This method aims to inhibit the activity of lipase, an enzyme responsible for breaking down fats and leading to rancidity. At temperatures below 3°C, lipase activity is significantly reduced, thus slowing down the process of oxidative rancidity. Research shows that storing rice bran under these conditions can reduce oil content to 6% after 50 days. This method is effective across a wide pH range of 10 –12.^34^ However, when stored at room temperature, lipase activity is no longer inhibited, causing a rapid increase in oxidative rancidity. Cold temperature treatment is not ideal for large-scale industrial food production due to the high costs and logistical challenges associated with maintaining consistently low temperatures, especially in environments with fluctuating conditions.^30^^,^^34^

##### Acidic treatment

Acidic treatment involves lowering the pH of rice bran using various acids to reduce lipase activity. Common acids used include hydrochloric acid, acetic acid, and propanol. Lipase activity in rice bran peaks at a pH of 7.5–8.0. By applying acidic treatments, the pH is lowered, thereby reducing the activity of lipase. Various chemicals, such as hydrochloric acid, phosphoric acid, acetic acid, and sodium metabisulfite, have been tested. Hydrochloric acid, at a dosage of 30 mL/kg, has been particularly effective in significantly lowering lipase activity during a 60-day storage period at room temperature.^35^

Acidic treatment is more feasible in regions where other methods, like heating, are impractical due to unreliable or insufficient electricity. It is generally more cost-effective than maintaining cold storage. The effectiveness of the acidic treatment has been demonstrated over a 60-day storage period at room temperature, making it a practical option for medium-term storage. Proper handling and dosage of acids are crucial to ensure safety and effectiveness. Both cold temperature treatment and acidic treatment offer viable solutions for stabilizing rice bran, but they cater to different needs and environments. The acidic treatment provides an adaptable and cost-effective solution, particularly in regions with less reliable electricity, making it a preferred method for stabilizing rice bran under various conditions.

#### Heating Process

##### Dry and moist heating

In a dry heating method, the FFA content can be reduced to 10% after 8 weeks of storage.^34^ Dry and moist heat treatments, including hot air heating, toasting, roasting, steaming, and autoclaving, are widely used for stabilizing rice bran. It was reported that hot air heating at 150°C for 10 minutes, roasting at 150°C for 2 minutes, and steaming at 130°C for 2 minutes significantly lowered FFAs, acid value (AV), and peroxide value (PV) compared with raw rice bran (RRB).^37^ Again, rice bran after dry heat with 20% moisture at 110°C for 5 minutes inactivates lipase/esterase activity. Rice bran with 10% moisture has also achieved the same inactivation with heating at 120°C for 40 minutes.^38^ Moist heating treatments, like steaming or pre-moisturization, are often more effective in stabilizing rice bran compared with dry heating methods, as evidenced by the superior results obtained with steaming, including minimal oil yield reduction and lower FFA content after 50 days.^38^ Achieving proper stabilization of rice bran depends on factors such as the initial moisture content, treatment duration, and temperature.^48^

##### Infrared heating

Infrared (IR) heating, inactivates lipase activity without changing the rice bran quality. The IR rays penetrate the rice bran and convert radiation energy to heat energy. With the IR heating of unprocessed rice at 60°C followed by 4–5 hours of temperature treatment, FFA content was reduced to less than 10% when compared with control for 7 days without altering the RBO quality.^44^ Similarly, Irakli et al^31^ found that lipase activity was reduced when rice bran was exposed to IR heating at 140°C for 15 minutes after 6 months of storage. The FFA content of both raw and IR-stabilized rice bran samples was monitored every 15 days over a 6-month storage period. Results revealed that rice bran stabilized at 600 W of IR power for 5 minutes maintained an FFA content below 5% for 165 days.^45^ Medium-wave IR heating demonstrated superior efficacy in limiting the escalation of FFA levels compared with short-wave IR heating under identical conditions of IR emitter power of 700 W and a duration of 3 minutes.^54^ The IR-vacuum technique showed optimal stabilization of rice bran under specific conditions: IR power at 999 W, treatment duration of 562 seconds, and vacuum pressure set to 650 mmHg. These parameters successfully reduced the FFA content to below 5% (3.29% as oleic acid) and maintained stability over a 60-day storage period at 35°C.^46^ The effects of IR radiation heat treatment on rice bran and its storage characteristics under various temperatures have revealed that subjecting the sample to IR radiation heating at 300°C for 210 seconds reduced lipase activity by 73.05% and peroxidase activity by 81.50%.^47^

##### Microwave heating

Stabilization through microwave heating reduces enzymatic activities, enhancing storage stability. Microwave treatments at higher power and longer durations result in lower levels of FFAs, AV, and PV during storage. Optimal stabilization is achieved at 925 W for 3 minutes, ensuring prolonged storage without significant rancidity.^48^ Microwave heat treatment effectively inactivates lipase and improves the shelf life of rice bran, preserving vital nutrients and ensuring safety and quality for various food applications. In another study, the optimal stabilization was achieved with 16% moisture content for 4 minutes, making it a recommended method for enhancing rice bran's nutritional value and usability.^55^ It was reported that microwave pretreatment (130–880 W for 0.5–5.0 minutes) increased antioxidant activity by 0.5-fold, total phenolic content by 1.3-fold, and total flavonoid content by 0.9-fold. Optimal conditions (440 W for 2.5 minutes) significantly boosted total tocols (2.6-fold), γ-oryzanols (1.6-fold), phytosterols (1.4-fold), trans-*p*-coumaric acid (10.3-fold), and kaempferol (8.6-fold). This method demonstrates the potential for enhancing rice bran's nutritional value and usability in various industries.^49^ Continuous microwave heating effectively stabilizes rice bran by reducing FFAs, PV, and 2-thiobarbituric acid (TBA) values during storage. Raw rice bran with 21% moisture was heated at 6400 W and 2450 MHz for 15.75 minutes, then stored at 4°C and 25°C, respectively, for 16 weeks. After this period, FFAs in RRB increased significantly, but no significant changes were observed in continuously microwaved rice bran. The PV and TBA values also remained lower in microwaved rice bran compared with RRB. This method prevents hydrolytic and oxidative rancidity, maintaining rice bran's stability and quality.^50^

##### Extrusion

Extrusion is an effective and one of the best stabilizing processes of rice bran, with high temperature and pressure that inactivate the lipase and peroxidase activities and help improve rice bran properties. The FFA content can be reduced in extruded rice bran more than in non-extruded rice bran, and more nutrients in rice bran are retained after extrusion. This method is very effective due to its high production efficiency, short time processing, and energy consumption as well as its simple operating system that gives the food product a unique size, shape, and physiochemical properties.^53^ The extrusion method stands out as a promising technique for stabilizing rice bran, effectively extending its shelf life and preserving its nutritional integrity. This method involves subjecting rice bran to high temperature and pressure conditions within an extruder, which can efficiently deactivate enzymes, such as lipase responsible for the degradation of lipids in the bran. It was reported that the impact of the extrusion treatment and varying temperatures (15°, 25°, and 40°C) on rice bran over 60 days resulted in significant changes in its storage stability, lipid oxidation, enzymatic activity, fatty acid composition, and protein characteristics. The report uncovered changes in rice bran's protein and amino acid compositions during extrusion and storage, with peroxidase and lipase activities decreasing significantly post-extrusion. However, PV, FFAs, and malondialdehyde content increased notably. Storage stability correlated positively with duration and temperature. The oleic acid/linoleic acid ratio remained stable, while total and essential/nonessential amino acid ratios decreased. First-order kinetics described enzymatic changes, with extrusion not affecting crude protein or essential subunits. Overall, optimized extrusion showed promise in stabilizing rice bran.^41^ The utilization of rice bran as feed in extrusion processes, ranging from 0% to 30%, along with controlled extrusion temperatures below 150°C and centrifugation speeds below 10 g, highlights the method's adaptability and effectiveness. Particularly for plant-based meat, a higher moisture content (60%–70%) is used, ensuring optimal processing conditions. Extrusion enhances hardness and bulk density while minimizing expansion.^56^ The most effective conditions for extruded rice bran were observed at temperatures ranging from 100° to 130°C. The lowest levels of lipase and peroxidase activities were achieved specifically at a die temperature of 123°C, centrifugation speed of 15 g, and an initial moisture content of 10.8%. Extruded rice bran has enhanced color and increased dietary fiber, while preserving key vitamins. Moreover, it has reduced phytic acid and vitamin E levels, making it a healthier ingredient. This stabilization method has improved water-holding capacity and reduced oil absorption, making it suitable for various food applications, indicating its potential for healthier food products.^42^ The incorporation of rice bran into extruded foods elevates their nutritional profiles, notably enhancing total phenolics, antioxidant activity, and functional properties, thereby fostering sustainability in food production. Ultimately, the extrusion method stands out as a promising avenue for crafting nutritious, functional, and sustainable food products enriched with rice bran.

##### Ohmic heating

Ohmic heating is the most viable process for rice bran stabilization procedures. This process involves an alternating current that is passed through the bran, generating heat from the electrical resistance of the bran.^28^ The major factor of effective ohmic heating stabilization is moisture content. If the moisture content of rice bran is 10%–20%, then the FFA content of the bran has been found to be approximately 4%.^30^ Furthermore, after 75 days of storage, the FFA percentage in ohmic-heated rice bran was determined to be 4.77%, but the FFA percentage in RRB without ohmic heating was 41.84%. According to Dhingra et al,^57^ in addition to FFA, the peroxide and AVs were also reduced. Total lipid extraction from ohmic-heated rice bran is 92%, whereas this is 53% in RRB. Therefore, ohmic heating with moisture addition has been used to effectively stabilize the rice bran as compared with a control. This method has been successfully applied for rice bran stabilization and increased oil extraction in the industry for making food items.^28^

The quality of the oil yield has been compared among the domestic heating stabilization methods, such as roasting, steaming, hot air drying, and microwave heating. Hot air drying, as well as microwave heating, are highly efficient stabilizing methods that extract higher yields by lowering FFAs, AV, and PV. However, when choosing between hot air drying and microwave heating, hot air drying is the most cost-effective option for large-, small-, and medium-scale operations in rural areas, while microwave heating is not.^37^ Each stabilization method offers unique advantages, such as reduced enzymatic activity, improved storage stability, and enhanced nutrient preservation. Optimal conditions vary depending on factors like moisture content, treatment duration, and temperature. Among the various stabilization methods, extrusion, microwave, and IR heating show significant promise for industrial-scale processing. Additionally, steaming and drying are commonly used in the RBO industry. It is essential to maintain the low moisture content in stabilized rice bran during storage to prevent the reactivation of lipases.^32^

#### Fermentation Process

There has been a significant amount of food resources wasted because rice bran has been mostly utilized for animal feed up to this point, as was mentioned earlier.^5^^,^^6^ Because of the harsh taste and unpleasant flavor that are brought about by lipid oxidation, the value of rice bran is negatively impacted.^42^ Therefore, rice bran's sensory attributes have been enhanced through the application of numerous food-processing methods, such as extrusion and fermentation. As nutrition and health become increasingly important, the effects of these processes on nutrients and phytochemicals are starting to receive more attention. Extrusion and microorganism fermentation can facilitate the breaking of covalent bonds through physical or biological activities that convert bound phenolics in cereals into free forms.^58^^,^^59^ Several nontoxic fungi and bacteria have been used in the fermentation of rice bran.^51^ Fungi such as *Rhizopus*, *Aspergillus*, and *Saccharomyces*^60–62^ and bacteria such as *Lactobacillus* and *Weissella*^63^^,^^64^ can be used to ferment rice bran. Seyoum et al^52^ also described that the key metabolites that have great nutritional value in gut health like arabinose, maltose, and essential amino acids and vitamins have been identified from the fermented rice bran by the bacterial probiotics such as *Bifidobacterium longum*, *Limosilactobacillus fermentum*, *Lacticaseibacillus paracasei*, *Lacticaseibacillus rhamnosus*, and *Escherichia coli* after the incubation with the yeast probiotic *Saccharomyces boulardii.* Fungi and bacteria that have been involved in fermenting the rice bran during food processing secrete several enzymes, such as amylase, β-glucosidase, protease, etc. These enzymes help change the rice bran constituents to other metabolites like sugar, alcohols, organic acid, amino acids, antioxidant compounds, etc, which improve the quality of the food. **Table 2**^62^^,^^65–73^ presents the fermentation methods involving yeast, fungal, and bacterial cultures applied to rice bran, emphasizing their role in disease control and prevention.

**Table 2.** Yeast, Fungal, and Bacterial Fermentation of Rice Bran for Disease Control and Prevention

Microbes	Reported bioactivity of fermented rice bran	References
*Saccharomyces cerevisiae*	Anti-stress, anti-fatigue, anti-inflammation	Kondo et al (2016)^62^ Kim et al (2002)^65^
*Aspergillus oryzae*	Anti-inflammation, antioxidant, protects against gastrointestinal damage	Onuma et al (2015)^66^ Punia et al (2021)^76^ Ochiai et al (2013)^67^
*Lactiplantibacillus plantarum*	Anti-inflammation, prevents cognitive impairment in rats	Kondo et al (2016)^62^
*Saccharomyces boulardii*	Reduced growth of human B lymphomas	Ryan et al (2011)^68^
*Lentinus edodes*	Anticancer	Kim et al (2007)^69^
*Rhizopus oryzae*	Antioxidant	Kupski et al (2012)^70^
*Lacticaseibacillus paracasei*	Antimicrobial, reduces salmonella growth	Nealon et al (2017)^71^
*Bifidobacterium longum*	Modulation of gut microbiome and colon cancer	Nealon et al (2019)^72^ Kumar et al (2022)^73^

## EXTRACTION OF RICE BRAN OIL

**Figure 3.**
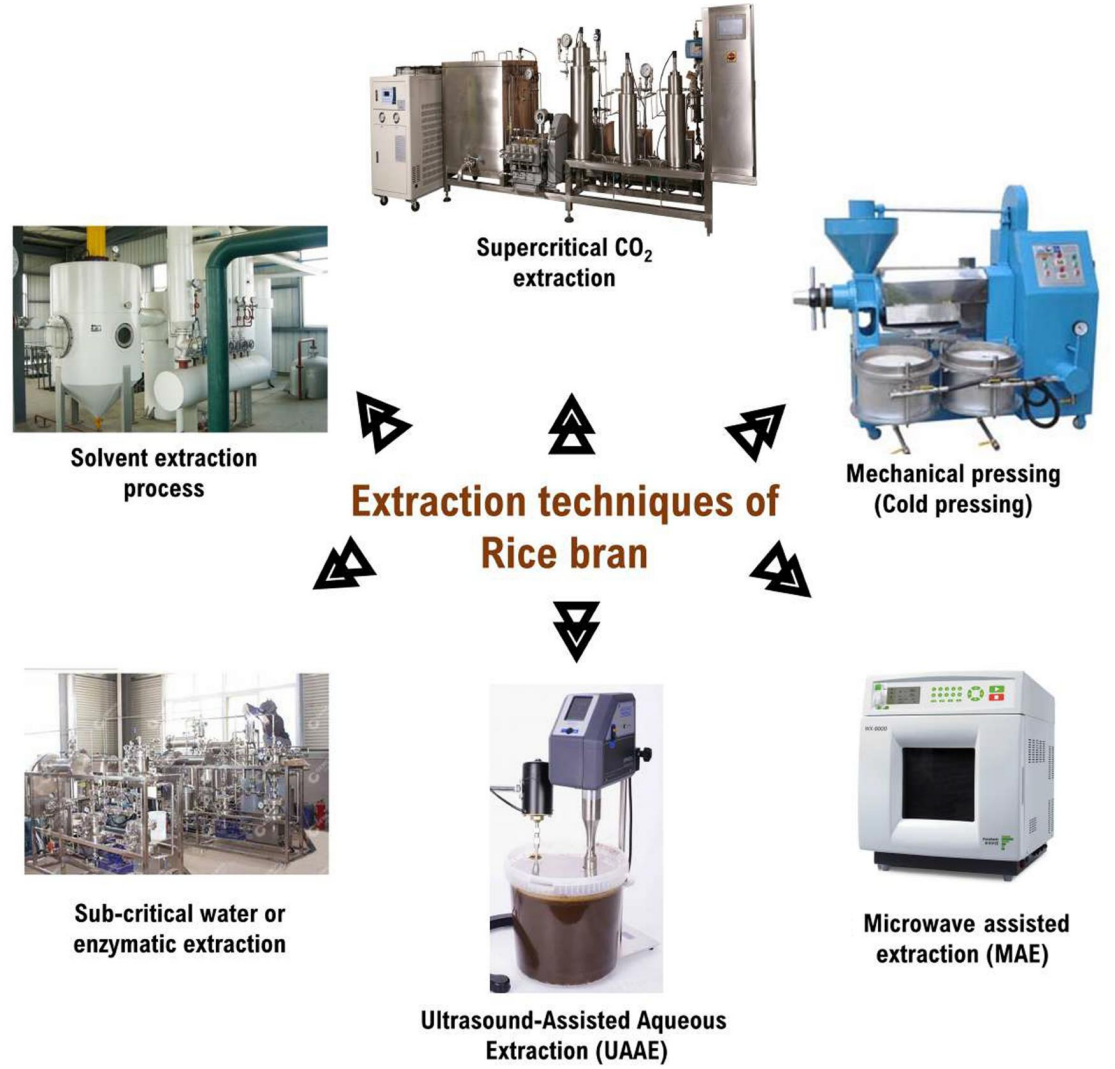
Different Methods and Instrumentation Technologies Utilized for Rice Bran Extraction. Solvent extraction (methanol, ethanol, hexane) utilizes a Soxhlet extractor or similar apparatus to dissolve oils and bioactive compounds. Mechanical pressing (cold pressing) uses a cold press machine to physically extract oil. Microwave-assisted extraction uses a microwave reactor to enhance extraction efficiency. Ultrasound-assisted aqueous extraction uses an ultrasonic bath or probe to disrupt cell walls and facilitate the release of bioactive compounds. Sub-critical water or enzymatic extraction involves a high-pressure reactor for sub-critical water extraction or a bioreactor for enzymatic hydrolysis. Supercritical fluid extraction utilizes a supercritical CO_2_ extractor to obtain pure extracts

Rice bran oil can be extracted from rice bran, typically via solvent extraction. Several solvents are used for extraction procedures, such as isopropanol, ethanol, methanol, acetone, and hexane, as well as in aqueous conditions or water. These traditional solvent extraction procedures are volatile and flammable and create air pollution due to industrial use. Out of these solvents, hexane extraction is very toxic to humans and not environmentally friendly.^74^ According to Fraterrigo et al,^75^ several new environmentally friendly innovative technologies have been developed by optimizing the pre-existing process, known as green extraction principles of nonconventional processes such as ultrasound, microwaves, superficial fluids, sub-critical extraction, etc. **Figure 3** and **Table 3**^76–83^ provide an encompassing visual overview of the diverse methodologies and instrumentation technologies used in rice bran extraction.

**Table 3.** Rice Bran Extraction Methods and Techniques

Techniques of extraction	Optimum condition	Inference or finding	References
Solvent extraction process by methanol, ethanol, and hexane	3 to 5 h of extraction time with 69°C, 78°C, and 65°C temperatures for hexane, methanol, and ethanol, respectively, followed by 1 h drying and 1 h vacuum condition	The highest yield has been found in ethanol solvent as compared with methanol and hexane, but low FFA has been found in hexane solvent extraction. A different impact has been reported in different solvent extractions.	Punia et al (2021)^76^ Wongwaiwech et al (2019)^78^
Mechanical pressing (cold pressing)	Temperature >50°C	Total nutraceuticals of the rice bran byproduct have been decreased by cold pressing extraction as compared with the solvent extraction process.	Wongwaiwech et al (2019)^78^
Microwave-assisted extraction (MAE)	Solid to liquid ratio, 0.42; power, 100.68 W; and time, 100.68 s	5–10-kDa fractionated rice bran protein has been found to have the highest ACE inhibition activity, DPPH, OH radical scavenging activity, and Trolox equivalent antioxidant activity.	Hayta et al (2021)^77^ Pandey and Shrivastava (2016)^79^
Ultrasound-assisted aqueous extraction (UAAE)	Sonication time, 60 s; sonication temperature, 40°C	The highest RBO has yielded 17.6%. Sonication time and temperature positively increased the extraction yields.	Gotama et al (2021)^80^ Djaeni and Listyadevi (2019)^81^
Sub-critical water or enzymatic extraction	Temperature, 120°C; time, 30 min	Milk analogs have been found due to protein extraction from defatted rice bran, which can be store at least for 1 mo at room temperature.	Wongthaweewatana et al (2021)^82^
Supercritical CO_2_ extraction	Temperature, 48.9°C; pressure, 23.9 MPa; and CO_2_ flow rate, 29.8 g/min	The highest yield of γ-oryzanol was found (ie, 36 000 mg/kg). Ethanol acted as a co-solvent or input factor.	Kayathi et al (2021)^83^

The efficiency and quality of rice bran extracts vary with each method. Solvent extraction provides high yields but may leave residues. Mechanical pressing preserves nutrient integrity but yields less oil. MAE and UAAE enhance extraction efficiency while being more environmentally friendly. Sub-critical water and enzymatic extraction offer selective and green extraction options. SFE delivers pure and high-quality extracts, ideal for specialized applications. The choice of method depends on the specific goals and requirements of the extraction process.

Abbreviations: ACE, angiotensin-converting enzyme; DPPH, 2,2-Diphenyl-1-picrylhydrazyl; FFA, free fatty acid; OH, hydroxyl radical; RBO, rice bran oil; SFE, supercritical fluid extraction.

### Conventional Extraction Process

#### Solvent Extraction

The solvent extraction method is best for the extraction of maximum oil from any type of low-oil-content materials for the pre-processing of food.^84^ Hexane extraction is the most common solvent used in RBO extraction because it is cheap, efficient, and best for oil extraction. The rice bran sourced from Afikpo, Abakaliki, and Emene was investigated using 3 solvents—chloroform, petroleum ether, and *n*-hexane—over a constant extraction time of 120 minutes via a Soxhlet extractor. The physicochemical properties of the extracted oil were evaluated, revealing a boiling point of 77°C. The study concluded that chloroform was the most effective solvent for extracting RBO, followed by petroleum ether and *n*-hexane, with the highest yield obtained from Abakaliki rice bran using chloroform.^85^ Moreover, in the comparison between oil-yielding quantity between hexane and isopropanol extract, isopropanol extraction gives a much higher yield compared with hexane. Hence, isopropanol has been used as an alternative solvent to hexane extraction from stabilized rice bran. Ethanol is the most environmentally friendly solvent used in the RBO extraction industry. Ethanol extraction has been used without heating and has given satisfactory results.^86^ Furthermore, approximately 80% of RBO has been yielded by using absolute solvents at 80°C in a single-stage bath, whereas the presence of water in the solvent significantly reduced the oil extraction.^87^ Ethanol is used for maceration in the extraction of RBO. The maceration method is accompanied by stirring. This method is considered safer for consumers as well as the environment.

#### Mechanical Pressing (Cold Pressing)

Cold pressing, also known as mechanical pressing, is an efficient and popular method for extracting oil from rice bran. This process is considered more advantageous than solvent extraction due to its simplicity, safety, cost-effectiveness, and labor-saving qualities. Cold pressing does not involve the use of heat or chemical solvents, making it a preferred choice for many industries aiming to maintain the natural integrity of the oil. The extracted oil retains greater nutritional value, as it is not subjected to high temperatures or chemical reactions that could degrade its quality.^76^ The process involves compressing the rice bran using mechanical and hydraulic pressing. Typically, the method includes the following sequential steps: initial cold treatment, mechanical compression with mild heat, followed by a filtering process. This sequence ensures the extraction of high-quality oil, although the yield might be slightly lower compared with solvent extraction methods. Cold pressing is widely adopted in rice bran processing due to its ability to preserve the natural flavor, color, and health benefits of the oil, making it suitable for various culinary and industrial applications.^34^ Its simplicity and cost-effectiveness make it an attractive option for both small-scale and large-scale operations, ensuring that the nutritional and bioactive compounds in the RBO are preserved.^88^

This extraction method preserves heat-sensitive vitamins and antioxidants, such as vitamin E, ensuring that the oil retains its nutritional profile.^89^ Cold-pressed RBO is ideal for culinary uses due to its rich flavor and nutritional value, making it suitable for salad dressings, drizzling over dishes, and light sautéing. In the cosmetics industry, its preserved antioxidants and vitamins provide moisturizing, anti-aging, and protective properties, while in the pharmaceutical sector, the retained bioactive compounds are valuable for formulations aimed at reducing cholesterol and providing antioxidant benefits. Cold pressing involves the use of a mechanical pressing with gentle heating, followed by filtration to obtain clean RBO. Pretreating rice bran at low temperatures before cold pressing can enhance the quality of the RBO.^90^ Overall, cold pressing enhances the health benefits, quality, stability, and natural flavor of RBO, highlighting its superiority over other extraction methods.^91^

### Nonconventional Extraction Processes

#### Microwave-Assisted Extraction

Microwave-assisted extraction (MAE) is one of the newly emerging technologies used for bran oil extraction. The short reaction time, low energy requirement, type of solvent use, solid to liquid ratio, and higher temperature favor a high yield of bioactive compounds as well as purity of the oil.^75^^,^^77^ Similarly, the innovative approach of 2 steps of MAE has obtained a superior quality of oil, such as the content of phospholipid, tocopherol, FFA, and antioxidant activity, as compared with solvent-extracted oil.^79^ Furthermore, MAE is safe, and the complete extraction procedure is carried out under atmospheric conditions.^76^ In the extraction of rice bran protein, a response surface methodology was utilized with MAE. The optimized conditions included using 1000 W of microwave power, an extraction time of 90 seconds, and a solid-to-liquid ratio of 0.89 g rice bran to 10 mL of distilled water. Comparing the results, the protein yield obtained from MAE was approximately 1.54 times higher than that with alkaline extraction.^92^ Another experiment with MAE was developed to enhance the production of RBO with more environmentally friendly solvents, such as ethanol and d-limonene, which were investigated as alternatives to *n*-hexane. The results demonstrated that the combination of d-limonene and MAE achieved the highest RBO yield (24.64%) as well as consuming less energy (95 Wh/g of oil).^93^

#### Ultrasound-Assisted Extraction

In the food-processing industries, the ultrasound-assisted extraction (UAE) process has been applied as an innovative extraction process as it satisfies the green chemistry principles. The advantages of UAE are that it is less time- and energy-consuming, consumes fewer solvents, and is cost-effective. The technique also produces highly pure active compounds and has been applied to the enhanced extraction of polysaccharides.^94^ Maximum RBO has been extracted by the UAE method using *n*-hexane as a solvent at an optimal temperature. The highest bran yield by UAE has been found to be 20.35% at 60°C with a bran-to-solvent ratio of 1:5 wt/vol.^73^ The UAE process has been proven to be a promising method that decreases the processing time as well as increases the extracted polysaccharide quantity by 4-fold as compared with hot water extraction when using a defatted rice bran to water ratio of 1:20 wt/vol with a temperature of 70°C for 20 minutes.^95^ This is similar to other studies that utilized processes that avoid heat and can help preserve the polysaccharide structure.^96^

Ultrasound-assisted extraction optimizes the extraction of phytochemicals, especially anthocyanins, from black rice bran. Using response surface methodology, it was determined that the optimized extraction conditions involve using acidified ethanol as the solvent and varying ultrasound power and extraction time.^97^ Exploring UAE conditions, a study focusing on optimizing oil extraction from rice bran found that higher power and longer treatment times increased oil yield but also impacted quality. Interestingly, UAE demonstrated results akin to pressurized liquid extraction, underscoring its potential for efficiently extracting oil from rice bran. Parameters such as ultrasound power (6.11, 12.23, and 18.35 W/cm^2^) and treatment duration (5, 15, and 25 minutes) were varied to assess their influence on extraction efficiency and oil quality.^98^

#### Sub-critical Extraction

Sub-critical fluid is known as pressurized or hot liquid solvents.^99^ Various types of solvents are used in this type of extraction technique, including hexane, but propane-butane is primarily used due to its low temperature, low pressure, colorless properties, no emission of toxic products, and being considered to be environmentally friendly. The advantage of this extraction technique is the use of low pressure and low temperature as compared with the superficial fluid extraction technique. It is a continuous process where solvents are removed at a low temperature by using a vacuum.^75^^,^^76^^,^^99^ Sub-critical fluid temperature influences the oil production from rice bran. It has been found that the most amino acids and organic acids were yielded from rice bran extraction by this technique and 127°C was found to be the optimum temperature for amino acids, whereas 190°C was found to be the optimum temperature for organic acids. It is an environmentally friendly technique and has successfully been applied for rice bran treatment and production of high value products.^100^ Similarly, the sub-critical carbon dioxide (CO_2_) extraction technique has been successfully used, which has higher oxidative stability and a higher level of compounds such as tocopherols, tocotrienols, oryzanols, and other FFAs in the extracted oil as compared with the traditional *n*-hexane solvent extraction technique.^99^^,^^101^

#### Supercritical Fluid Extraction

When a certain fluid is subjected to above-critical temperature and pressure, such fluid is known as a supercritical fluid. There is no risk of contamination by a solvent or any other chemical modification problem in the supercritical fluid extraction (SFE) method.^99^^,^^102^ Moreover, by applying optimized handling parameters such as temperature, pressure, and CO_2_ flow rate, γ-oryzanol compounds have been extracted by the supercritical-CO_2_ (SC-CO_2_) extraction technique.^83^ Similarly, the combination of a liquid solvent, such as expanded hexane with CO_2_, has extracted a high yield of bio-oil under specific conditions.^103^ The bran of various rice cultivars, sourced from Portugal, was utilized to investigate the SC-CO_2_ extraction conditions for obtaining RBO. Several key extraction parameters, including plant loading (10–20 g), CO_2_ flow rate (0.5–1.5 L/min), pressure (20–60 MPa), and temperature (40°–80°C), in the extraction process were involved. Additionally, the antioxidant activity and fatty acid composition of the diverse rice bran varieties were evaluated and compared. Supercritical-CO_2_ is a good method for RBO extraction, contributing to the valorization of RB, hence enhancing the transition to a sustainable economy. Higher pressure (up to 40 MPa) increased the yield, but temperature (40° to 80°C) had less impact. The rice cultivar Indica Sirio had the highest yield (15.60%), while Japonica Euro had a lower yield (11.48%).^104^

Sub-critical extraction uses lower pressure and temperature than SFE, making it less energy-intensive but potentially yields lower extraction efficiency and quality. For industrial use of RBO or rice bran products, the choice between SCE and SFE depends on several factors. Sub-critical extraction, utilizing conventional organic solvents at temperatures and pressures below their critical points, offers simplicity and cost-effectiveness, making it suitable for a wide range of applications. However, SFE, which uses CO_2_ in its supercritical state, offers advantages such as higher extraction efficiencies, selective extraction of target compounds, and minimal solvent residue, albeit requiring specialized equipment and being relatively more expensive. Considering the potential benefits in terms of extraction efficiency, selectivity, and purity, SFE may be preferred for industrial applications requiring high-quality RBO or products.

#### Nonconventional Processing Comparisons

Each extraction technique offers unique advantages. Microwave-assisted extraction provides high yields with shorter reaction times and lower energy expenditure, while UAE is known for its green chemistry principles, requiring less time, energy, and solvent. Sub-critical extraction utilizes low temperature and pressure, yielding amino acids and organic acids effectively. Supercritical fluid extraction, utilizing above-critical temperature and pressure, ensures minimal contamination risk and allows for the extraction of specific compounds such as γ-oryzanol. Each method has its own merits and suitability depending on the desired outcome and application.

## RICE BRAN FOR DEVELOPMENT OF FUNCTIONAL FOODS

Rice bran is the byproduct of rice that is separated from the rice in the rice-milling process, and the bran is traditionally used as livestock feeds (eg, chickens, cattle, pigs). In many cases, it is also discarded as waste. Due to the nutrient content of rice bran, it is now used in several food-processing industries through enzymatic treatment and fermentation processes.^105^ Rice bran is relatively simple to handle due to its adequate availability and it has low cost and nutrient density. The beneficial effects of rice bran are identified by its bioactive ingredients, including proteins, polysaccharides, fats, oils, and other micronutrient elements.^106^ Rice bran is considered a functional food due to its composition of prebiotic fibers and compounds such as tocopherol, adenosine, ferulic acid, γ-oryzanol, and tocotrienol.^5^^,^^107^ Rice bran can be fermented by distinct microbiota to develop bioactive compounds that can help in preventing disease via multiple mechanisms while increasing nutrient bioavailability.^8^

**Figure 4.**
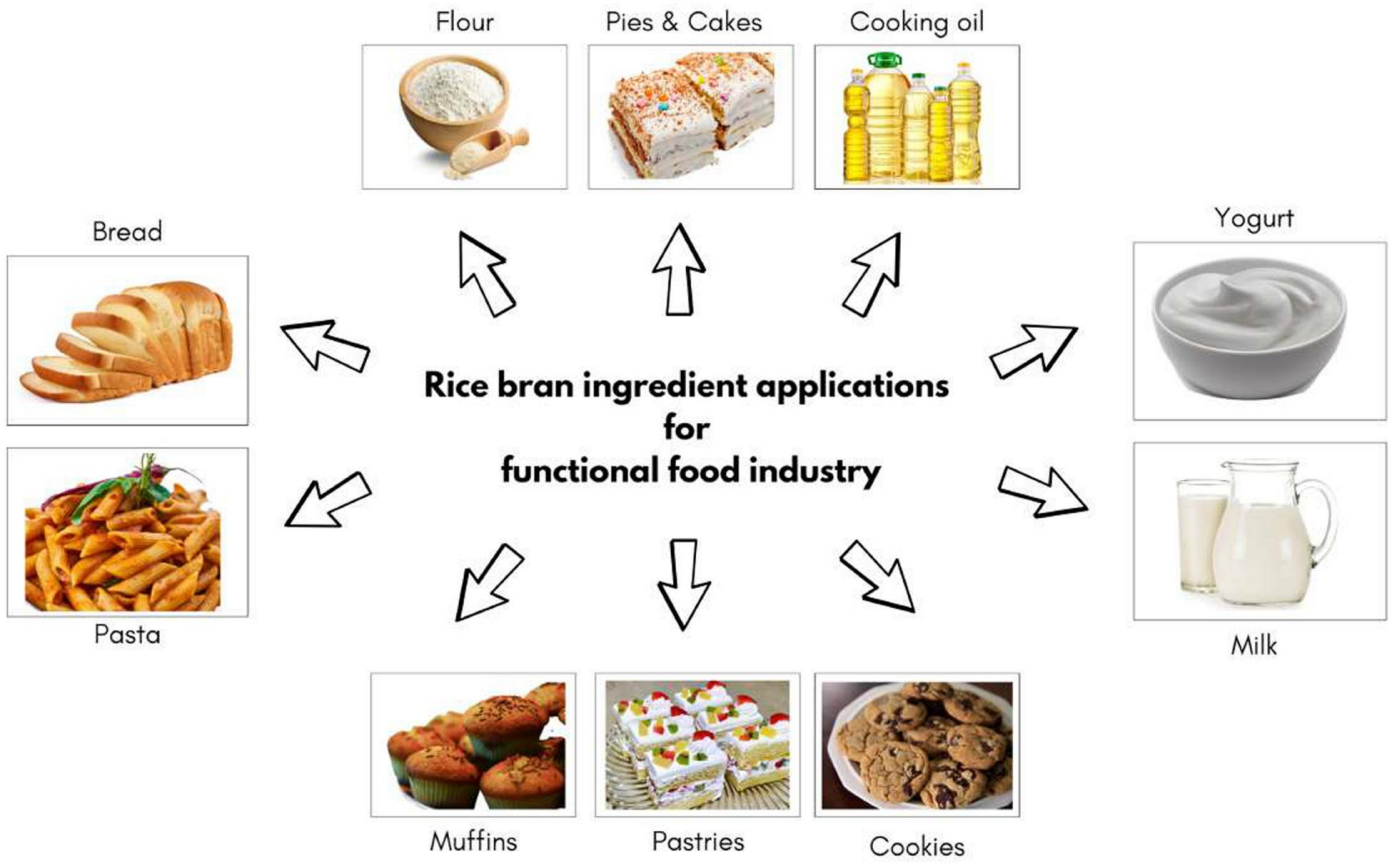
Applications for Rice Bran as a Food Ingredient. There are diverse applications of stabilized rice bran across multiple industries, emphasizing its role in enhancing nutritional quality, promoting health, and improving quality of life on a global scale. Rice bran’s versatility as an ingredient supports daily human dietary intake alongside sustainable and innovative healthier food-products

Rice bran fibers are important for reducing the risk of lifestyle-related diseases.^108^ Therefore, rice bran as a functional food can help in reducing several disease risks such as cardiovascular disease, chronic disease, cholesterol, and cancer.^76^^,^^109^ Rice bran has great potential to be used in several food types such as bread, cakes, noodles, muffins, cooking oil, milk products, pasta, ice creams, etc. **Figure 4** illustrates the diverse uses of rice bran as an essential component in a range of food categories and sectors, highlighting its extensive influence on daily diets globally and its potential to improve quality of life. Rice bran is an ideal ingredient for baking products due to its high-fiber content that enhances the regularity of stool in human consumption. Rice bran flour and RBO have been used for their rich protein content. Baked goods such as cookies, bread, crackers, pancakes, muffins, and other pastries with the use of rice bran have greater nutritional value as compared with other cereals.^105^ Rice bran oil has a high nutritional quality ideal for nutraceutical products such as snacks. It has been used for its notable antioxidant properties, and food items with RBO have storage stability. Pizza and pasta with stabilized and extracted rice bran flour have more bioactive functional properties as compared with other flours.^48^ Rice bran oil has recently replaced traditional cooking oil due to its pleasant smell and is used in baking foods because of its high oxidative properties. In addition to the high antioxidant property of RBO, this oil has shiny, appetizing characteristics due to the its retention on the surface of food.^109^^,^^110^ Despite several roles of RBO, it is also used in mixing with milk powder to stabilize and prevent deterioration of milk. Due to the high antioxidative properties of RBO and its rich vitamin E content, it helps in improving the oxidation stability of low-heat milk powder.^111^ Moreover, the addition of RBO with milk-based products can lead to the development of different innovative food items. Rice bran wax can be used as an alternative coating for fruits and vegetables, forming lipid-based edible coatings after refining.^112^ Pigmented rice varieties, such as red, black, purple, and brown rice, have color intensity related to bioactive constituents, giving the rice vibrant yellow, red, and purple colors. Different food colors were produced from the pigmented rice varieties and used as colorants for snacks, beverages, meat products, and sauces. The pigmented dye of rice bran is capable of coloring other different food items.^113^ Overall, rice bran as a byproduct that helps in several food preparations and has different disease-prevention properties. The use of rice bran in functional foods can lead to a healthy life.

## VALUE-ADDED FOODS WITH IMPROVED RICE GENETICS/CULTIVAR DEVELOPMENT

Improvement in rice genetics and cultivar development could also be a step towards the creation of value-added foods. Scientists have developed different genetic techniques to enhance the nutritional composition and functional properties of rice bran by selective breeding, genetic modification using the CRISPR/Cas system, allele mining, metabolomics, transcriptomics analysis, etc.^114^ The goal is to develop cultivars with elevated levels of bioactive compounds, such as different antioxidants, vitamins, minerals, and dietary fibers within the bran. Biofortification of rice bran refers to the process of enhancing the nutrient content through breeding and genetic techniques. Biofortification is sometimes helpful to improve the essential vitamins, minerals, and other beneficial compounds in rice bran, making it more nutritious and beneficial for human consumption. Normally, biofortification focuses on an increasing amount of the availability of nutrients like zinc, iron, vitamin A, and other vitamins and minerals that are typically lacking in conventional rice varieties. These advancements pave the way for the production of rice bran–based products and food items such as cookies, muffins, pizza, pasta, bran oil, milk, etc, that offer improved health benefits and high nutritional value to the consumers.^5^^,^^115^

The application of genetics and cultivar development for enhancing rice bran nutraceutical potential may involve specific genes regulating the synthesis or breakdown of bioactive components. Novel rice genetic lines can achieve desirable functional food attributes, as recently described for a suite of Asian rice varieties.^116^ Rice cultivars with the desirable trait can be developed by optimizing nutritional profiles and co-production of γ-oryzanol, dietary fiber, phytic acid, isovitexin, ferulic acid, γ-amino butyric acid, tocopherol, and other antioxidants.^117^ Korean rice landraces were found to be rich in γ-oryzanol components.^118^ These cultivar developments with genetic variation showed that black rice, particularly Indonesian cultivars, contain diverse phytochemical variants like anthocyanin and oryzanol, making it a promising functional food with potential nutraceutical properties.^119^ In addition to developing cultivars with improved properties, such as stability, flavor, and texture, rice bran can be incorporated into a diverse array of value-added foods. The versatility of rice bran, coupled with advancements in genetics, offers opportunities for innovation in healthier food options that replace ultra-processed food consumption. Notably, the antioxidant-rich characteristics of rice bran from genetic improvements could also assist in prolonging the shelf life and market value.

To enhance the vitamin and mineral content in the grain, multiple breeding experiments exist^120^ and cross-breeding may become the most appropriate approach. Biofortification of lipid-soluble vitamin E (ie, tocotrienols, T3s) that contain an isoprenoid side chain has been reported.^121^ T3s have several nutraceutical applications for use with regard to antioxidant, anti-inflammatory, anti-angiogenesis, and cardiovascular activities.^122^ Furthermore, limiting some trace elements such as arsenic is important. Published findings by Weber et al^123^ reported that arsenic concentrations vary in rice bran across environments and varieties. Concentrations of arsenic in rice bran differed by country of origin and by agricultural irrigation techniques. A human health risk assessment was also performed to identify safe consumption doses of daily rice bran supplementation for infants. Importantly, the Nicaragua and Mali infants who consumed rice bran daily for 6 months did not show any differences in stool or blood arsenic concentrations when compared with the control group.^124^ Overall, the integration of genetics and cultivar development in rice bran holds immense potential to produce value-added foods that not only contribute to a healthier diet but also offer a wider range of choices for consumers in the food market.

## ENHANCEMENT OF PHYTOCHEMICALS THROUGH PLASMA TECHNOLOGY

Plasma technology is an innovative approach to enhance the phytochemical content of rice bran. It is an environmental friendly technique and is used in many fields of science.^125^ Plasma, a partially ionized gas, is utilized to create a controlled environment in which physical and chemical reactions occur.^126^ It can increase the concentration of phytochemical compounds such as phenolics, flavonoids, and other antioxidants, which contributes to the health-promoting properties of rice bran. It has enhanced the extraction process for polyphenols from rice and maize bran using both atmospheric and vacuum cold plasma (CP). The resultant phytoextracts not only exhibited heightened antioxidant activity but also demonstrated superior in vitro digestion, elevated anti-inflammatory responses, and overall enhanced quality when compared with conventional extraction methods.^127^ In a separate investigation, Mehta et al^128^ applied CP to extract xylo-oligosaccharides from the dietary fibers of rice and corn bran. Mirroring the outcomes observed with polyphenols in their earlier study, the extracted xylo-oligosaccharides displayed improved gastric digestion and elicited anti-inflammatory responses without any cytotoxic effects on RAW 264.7 and HepG2 cell lines.^128^ Plasma treatment can further improve the bioavailability and solubility of these phytochemicals, enhancing their absorption and utilization in the human body. This atmospheric pressure system is used in various industries to inactivate microorganisms in various food products.^129^ It can eliminate various harmful microbes from the rice bran. Cold plasma treatment has been applied in oats sprouts and their nutritional value has been successfully enhanced.^126^ Due to the implementation of CP technology postharvest, the quality of rice grains has improved in Malaysia.^130^

## CONCLUSION

Rice bran emerges as a powerful functional food ingredient in the global food chain with diverse stabilization techniques, offering significant potential for enhancing nutritional contents, biofortification, and nutraceutical development. Despite challenges of rapid degradation during processing that limits shelf life, the solvent extraction, enzymatic treatments, and novel stabilization methods can preserve and even enhance the bioactive component profile. These multiple approaches are feasible to facilitate biofortification, and innovative extraction techniques for RBO hold promise for a range of health benefits. Collaborative efforts across countries, from agriculture to human studies, are essential to address reducing food waste of rice bran. There is a need to ensure sustainability of rice processing methods that maintain or enhance the nutritional value of the bran. While these stabilization techniques can be adapted to diverse environmental settings, special attention is warranted for the inclusion of dietary rice bran in food-production systems. This comprehensive review of rice bran stabilization methods and approaches supports the need for continued research and development in different countries to fully harness rice bran's potential as a functional food ingredient, and to leverage cutting-edge technologies to address global nutrition challenges.
